# Quality of engagement with in-the-moment digital CBT during periods of distress: A study of patients with suicidal thoughts and behaviors

**DOI:** 10.1016/j.jad.2026.121248

**Published:** 2026-01-28

**Authors:** Molly I. Ball, Matthew J. Flics, Joseph S. Maimone, Stuart Beck, Michelle B. Stein, Matthew K. Nock, Kate H. Bentley, Evan M. Kleiman

**Affiliations:** a Department of Psychology, Rutgers University, 53 Avenue E, 6^th^ floor, Piscataway, NJ, 08854, USA; b Department of Psychology, Harvard University, 33 Kirkland St, 12^th^ floor, Cambridge, MA, 02138, USA; c Department of Psychiatry, Massachusetts General Hospital, 185 Cambridge Street, 2^nd^ floor, Boston, MA, 02114, USA; d Department of Psychiatry, Harvard Medical School, 25 Shattuck Street, Boston, MA, 02115, USA; e Department of Psychological and Brain Sciences, Boston University, 64 Cummington Mall # 149, Boston, MA, 02215, USA

**Keywords:** Ecological momentary intervention, Suicidal thoughts, Emotion regulation, Cognitive behavioral therapy, Skills practice, Engagement

## Abstract

Practicing CBT skills outside of therapy sessions is a core component of effective treatment, yet meaningful engagement can be difficult during periods of elevated psychological distress. Smartphone-based ecological momentary interventions (EMIs) offer a promising approach to support real-time skill use, but most research has focused on frequency rather than quality of engagement. This study examined the quality of CBT skills practice in a 28-day smartphone-based EMI following inpatient discharge and explored its associations with internal context and intervention effectiveness. Twenty-three adults hospitalized for suicidal thoughts and behaviors participated, selecting and practicing one of three guided CBT skills exercises (Mindful Emotional Awareness, Countering Emotional Behavior, or Cognitive Flexibility) up to three times daily. A structured coding system was used to rate the completeness and relevance of 945 total exercises (0–2 scale). Quality varied primarily within individuals and was lower during moments of high suicidal intent and urge. When combining across all skills, quality was not associated with proximal change. However, higher-quality Countering Emotional Behavior practice predicted greater reductions in suicidal intent, and higher-quality Countering Emotional Behavior and Cognitive Flexibility practice predicted greater reductions in anger. These findings suggest that momentary distress can impact engagement quality, and that the short-term impact of skills practice may depend on both the specific CBT strategy and type of distress targeted. Understanding and enhancing engagement quality is a key step toward optimizing mobile interventions for individuals in the midst of psychological distress.

Cognitive Behavioral Therapy (CBT) helps patients identify and challenge distressing and maladaptive thoughts, emotions, and behaviors (Wenzel, 2017). CBT can effectively treat a wide range of psychiatric disorders and symptoms, including depression and suicidal thoughts and behaviors (Tarrier et al., 2008; Wu et al., 2022; Cuijpers et al., 2025). A key component of CBT’s efficacy is the regular practice of learned skills outside of therapy sessions, with longstanding research showing that higher compliance with CBT skills practice between sessions is an important variable in predicting improved outcomes (Neimeyer and Feixas, 1990; Mausbach et al., 2010; LeBeau et al., 2013; Kazantzis et al., 2016). Engaging in skill practice can be particularly challenging during extended periods of elevated suicide risk, such as during the month following psychiatric inpatient hospitalization, which is often full of stressful readjustment to life outside of the hospital. This is also the time when use of such skills may be most crucial (Chung et al., 2017; Goldacre et al., 1993). During this critical period, barriers such as heightened emotional distress, diminished motivation, and challenges associated with the transition back into the community may interfere with an individual’s ability to effectively engage in CBT-based interventions (Mutschler et al., 2019).

Smartphones and mobile health tools are promising solutions for bolstering skill practice by delivering brief, in-the-moment interventions (e.g., reminders to practice skills and guided instructions) and providing immediate, accessible support during the post-discharge period. Over the last decade, these ecological momentary interventions (EMIs) have become increasingly popular, and have been tailored to effectively address suicide risk during the post-hospitalization period by incorporating emotion regulation skills (i.e., skills aimed at managing intense negative affect and addressing cognitive inflexibility), as well as coping skills and safety planning strategies across different levels of suicide risk – an approach similar to the EMI examined in this study (Balaskas et al., 2021; Bentley et al., 2025; Jiménez-Muñoz et al., 2022; Kleiman et al., 2024). As these interventions become more commonly studied and applied, it is important to understand the nature of engagement with EMIs (i.e., *how* individuals engage) and how these factors may influence intervention effectiveness.

Although previous studies have examined the positive relationship between *quantity* of skills practices completed and treatment outcomes in outpatient CBT treatment as discussed above, the *quality* of skills practice (e.g., degree of meaningful effort and accuracy) may play an equally critical role in intervention effectiveness. For example, a patient practicing CBT skills five times a day may not benefit as much as a patient practicing only once daily if the latter engages more thoughtfully and deliberately. Indeed, a meta-analysis by Kazantzis et al. (2016) demonstrated that both quantity *and* quality of CBT skills practice influence treatment outcomes. This relationship between quality of skills practice and treatment outcomes remains under-explored in both EMI studies and studies focusing specifically on patients at high risk for suicide. Given the potential for EMIs to enhance access to CBT-based interventions, by providing guided skill practice to individuals who may not have regular in-person care, who need skills in certain moments, or who want to practice skills more independently, understanding the role of skill practice quality is crucial for optimizing their effectiveness.

This study addresses this gap by examining how the quality of EMI CBT skills practice relates to affective context and intervention outcomes in a high-risk population. Leveraging data from a recent EMI study designed to promote real-time emotion regulation CBT skill use following psychiatric inpatient hospitalization, we investigate the associations between affective context, skills practice exercise quality, and proximal intervention efficacy. First, we provide a descriptive analysis of skills practice exercise quality overall and for differences between skill types. Second, we test the associations between pre-EMI internal states and quality of skills practice exercise engagement. Third, we examine whether skill practice exercise quality is associated with intervention effectiveness, specifically proximal reductions in suicidal thoughts and related emotions.

## 1. Method

### 1.1. Participants

Participants were 23 adults, drawn from a larger sample of 26 adults (Kleiman et al., 2024), admitted to the inpatient medical psychiatric unit at Massachusetts General Hospital in Boston between August 2019 and April 2020. All participants were admitted to the hospital for suicidal thoughts and behaviors, were 18 years or older, had access to a smartphone, were fluent in English, and were willing to provide at least one collateral contact. Prior to being approached by study staff, each participant was also deemed capable of providing consent and appropriate for study participation by their clinical provider on the unit. Patients who had any factors which impaired their ability to consent or effectively participate in the study (i.e., cognitive impairment, acute psychosis, active withdrawal, severe or dangerous behavioral dysregulation) were excluded from participation. All participants provided informed consent prior to participation. Three participants were excluded due to not providing any EMI data. Demographic information for participants is in Table 1.

### 1.2. Procedures

At the earliest opportunity following admission to the inpatient unit, all participants reviewed and signed the consent document with study staff. Upon enrollment, participants downloaded a smartphone-based application (LifeData), received an orientation from study staff on how to use it, and had the opportunity to ask questions. Once set up, participants were prompted daily via the smartphone application to complete six brief ecological momentary assessment (EMA) surveys during both the inpatient and post-discharge study periods. Surveys were delivered randomly within a specified window tailored to each participant’s sleep schedule. Each survey remained active for 2 h, and surveys were sent at least 2 h apart so that only one survey could be accessed at a time. Each survey was comprised of questions asking participants to rate their current suicidal intent, suicidal urge, and relevant affect states (including agitated, angry, fatigued, positive, negative, grateful, connected, hopeless, and burdensome) on a 0–10 visual analog scale.

While on the inpatient unit, participants completed three one-on-one, 30-minute intervention sessions with a trained study therapist. Each session focused on one of three key CBT skills drawn from the Unified Protocol for Transdiagnostic Treatment of Emotional Disorders (Barlow et al., 2020). The first skill, Mindful Emotional Awareness (MEA), involves using present-focused emotional awareness to identify thoughts, feelings, and behaviors in the current moment without judgment. The second skill, Countering Emotional Behaviors (CEB), involves identifying current unhelpful emotional behaviors or urges, and alternative, more adaptive, behaviors. Lastly, Cognitive Flexibility (CF), involves identifying emotion-driven automatic thoughts and generating corresponding alternative interpretations. All intervention materials were developed by doctoral-level principal investigators based on existing Unified Protocol therapy materials. All study therapists completed mock sessions prior to delivering the intervention to ensure consistent coverage of the manualized content. During therapy sessions, participants were introduced to, and practiced, each skill, and were taught how the skills would relate to the EMI prompts they would later receive.

For the 28 days following discharge, participants continued to receive six daily surveys on their smartphone. Each day, three out of these six surveys included an intervention (i.e., EMI) component, where participants could choose to practice any one of the three CBT skills (MEA, CEB, or CF) after answering questions about the intensity of their in-the-moment suicidal thoughts and affect. After selecting a skill, participants were then guided through practicing it via step-by-step exercises which included instructions and text boxes to write responses (intervention launched in Qualtrics from LifeData). For example, if practicing CEB, participants would be prompted to identify and type in 1) current or recent distressing emotion(s), 2) any unhelpful emotion-driven behavior(s) or urges, 3) potential alternative actions, and 4) short-term and long-term effects of alternative actions (see Appendix A for EMI example images). If they completed the brief skills practice exercise, participants were then presented the same momentary suicidal thought and affect items as before the exercise. In addition to the three daily prompted skill practices (84 total across the 28-day post-discharge study period), participants could also self-initiate an EMI survey at any time to practice skills outside of prompted moments. During the other three surveys each day, participants were only asked to complete the momentary suicidal thought and affect items once, and did not receive any intervention prompts. Participants were compensated $0.50 for each EMA survey they completed, regardless of whether the survey contained EMI, and received a $2 bonus each day if they completed four or more surveys that day.

All study procedures were approved by the Institutional Review Board at Harvard University (protocol #IRB18-1813) with IRB reliance agreements with other involved institutions and adhered to all laws and institutional guidelines. This trial was registered before data were collected on ClinicalTrials.gov (NCT03950765).

### 1.3. Data analysis

#### 1.3.1. Quality coding

Quality of engagement with skills practice was operationalized as the completeness and relevance of participants’ responses to the guided skills practice exercises. We developed a structured coding template to rate the quality of each skills practice exercise (see Appendix B for details). Each skill was rated for quality on two key parts of the skill practice exercise, both on a 3-point scale: 0 (missing, limited, or irrelevant), 1 (somewhat relevant or complete), or 2 (highly relevant and complete). For example, in a CEB practice exercise, participants were rated for first being able to identify any unhelpful emotion-driven behavior(s) or urges and rated separately for being able to identify a helpful alternative behavior. When asked to identify an unhelpful behavior, no response or an irrelevant response (e.g., *“Be happy”*) would be rated 0. A relevant experience but not a specific behavior, such as *“Feel depressed”*, would be rated 1. A response that accurately identifies an unhelpful behavior, such as *“I might drink or cut myself”*, would be rated 2. The quality coding template can be found in Appendix B. For each skill practice exercise, the two quality ratings were averaged to create an overall quality rating on a scale from 0 to 2.

To ensure reliability of quality ratings, four coders (2 trained bachelor-level research assistants and 2 doctoral-level investigators) each coded two sets of 35 skills practice exercises and discussed code decisions for each. Then, a set of 260 randomly selected skills practice exercises (out of the 945 total) were double coded between the four coders, with an initial agreement rate of 67%, resulting in moderate interrater reliability (Kappa = 0.43, *p* < 0.001). An additional set of 300 exercises were then double coded by two bachelor-level coders with agreement rising to 83%, resulting in strong interrater reliability (Kappa = 0.67, *p* < 0.001). All codes with inter-rater disagreement were discussed and resolved with a doctoral-level investigator in discrepancy review meetings. Each of the 315 remaining entries was then coded by one trained and supervised bachelor-level research assistant.

#### 1.3.2. Statistical analyses

Overall quality of skills practice exercises was assessed using the *psych* and *dplyr* packages in R. A paired *t*-test was used to assess within-person quality differences between most-used and least-used skill types. ANOVA and subsequent Tukey’s HSD tests were run to assess differences in overall quality ratings between skill types. For assessing overall quality variation within participants, a mixed-effects model was run using the *lmer* function from the *lme4* package in R to calculate the intraclass correlation coefficient (Bates et al., 2015). To test associations between affective states, skills use, and outcomes, multi-level models were run using the *lmer* function from the *lme4* package in R. Models tested the associations between the intensity of specific momentary suicidal thought and affect states captured with EMA immediately prior to each skills practice exercise and quality of the time-matched skills practice exercise. A separate model was run for each affective state. In each model the predictor was momentary affect or suicidal thoughts and the outcome variable was quality. All variables were within-person mean-centered for interpretability using the *EMAtools* package in R. Multi-level models also examined whether quality ratings were associated with pre- to post-EMI change in suicidal thoughts and momentary affect. In these models, the predictor and outcome variables were flipped from the prior models described above. All models included a random intercept for each participant, accounting for repeated measures within individuals.

## 2. Results

### 2.1. Descriptive statistics

**Fig. 1.**
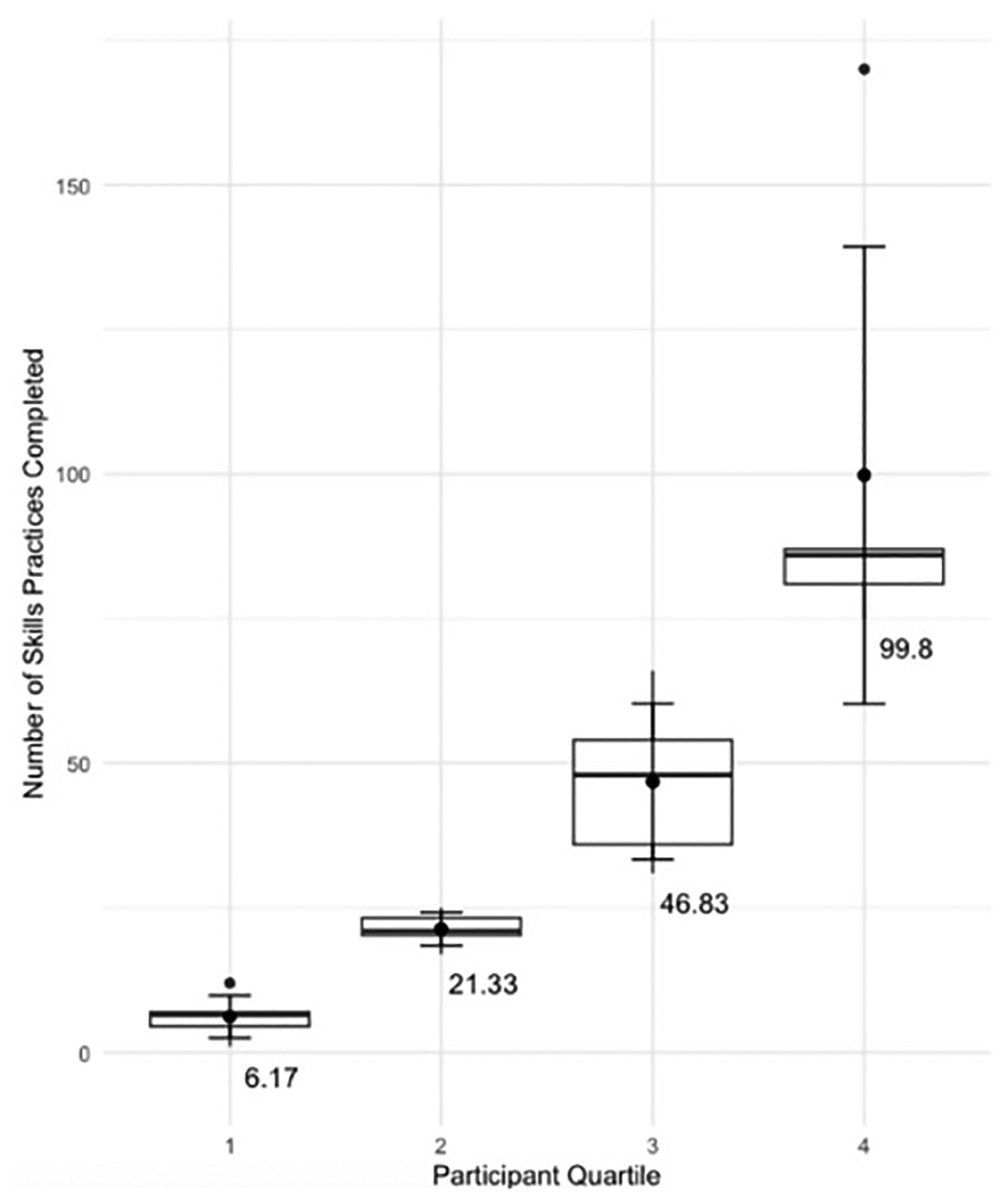
Number of skills practice exercises completed by participants, shown by quartile distribution. Averages for each quartile are indicated. Quartiles 1–3 each include 6 participants, and quartile 4 includes 5 participants.

In total, 945 skills practice exercises were completed by participants (*N*_MEA_ = 329, *N*_CEB_ = 241, *N*_CF_ = 375). Individual participants (*N* = 23) completed an average of 41 skills practice exercises each (*SD* = 39.52, range = 1–170). Fig. 1 shows the number of skills practice exercises completed by participants when divided into quartiles. Across all skills practice exercises, participants earned an average quality rating of 1.62 (*SD* = 0.31, range = 0.75–2.00). Across all skill practice exercises, 60% had a quality rating of 2 and the overall mean quality rating was 1.67 (*SD*: 0.49). The intraclass correlation coefficient (ICC) for quality ratings was 0.29, indicating that 29% of the variance in quality ratings is attributable to differences between participants and suggesting that quality of engagement fluctuated mainly within-person. Within participant, quality of engagement did not significantly differ between the skill they chose to practice the most often and the other two, less frequently practiced, skills (t(17) = −0.56, *p* > 0.05). By skill, MEA practice exercises had an average rating of 1.71 (*SD* = 0.44), CEB practices had an average quality rating of 1.74 (*SD* = 0.47), and TF had an average quality rating of 1.58 (SD = 0.54). Quality of skills practice exercise was significantly higher for both MEA and CEB when compared to TF (F = 9.27, df = 2, *p* < 0.001). There was no significant difference in the overall quality of skills practice exercises between MEA and CEB (*p* > 0.05).

### 2.2. Distress and skills practice

Pre-EMI intensity of suicide urge and intent were significantly negatively associated with subsequent skills practice exercise quality (*ps* < 0.05, Table 2). Pre-EMI intensity of affect states, however, was not significantly associated with skills practice exercise quality (*p* > *0*.05, Table 2).

### 2.3. Skills practice and clinical improvement

When combining across all three skills, skills practice exercise quality was not associated with proximal change pre- to post-intervention in any suicidal thought or affect states (*p*s > 0.05, Table 3). However, models testing each of the three skills separately found that higher quality of CEB was associated with greater proximal reductions in suicidal intent (*p* < 0.05, Table 3). Additionally, higher quality of both TF and CEB was associated with greater reductions in anger (*ps* < 0.05, Table 3). Quality of MEA skills practice exercises was not associated with proximal changes in any suicidal thought or affect states (*p*s > 0.05, Table 3).

## 3. Discussion

This study examined the quality of CBT skills practice exercises delivered through an EMI among individuals at high risk for suicide during and immediately following inpatient hospitalization. It further explored how engagement quality varied based on affective context and whether engagement quality was subsequently associated with proximal intervention efficacy. Our findings suggest that quality of engagement with the skills practice exercises in an EMI was high overall. While quality of engagement mostly fluctuated within individuals, findings show that quality of engagement varied slightly based on individual differences (i.e., some participants consistently engaged with higher-quality practice than others) as well as across different CBT skills. Additionally, severity of participants’ suicidal thinking (both suicide urge and intent) at the time of EMI delivery was associated with lower quality of their skills practice. Finally, skills practice quality was only selectively linked to proximal EMI efficacy, with unique effects observed for specific skills, not across all CBT skill types.

Overall, the high average quality of skill practice exercises indicates that individuals generally understood and engaged thoughtfully with the digital intervention. Within-person differences accounting for the variability of overall quality ratings, rather than between, suggests that engagement quality was not driven by person-level differences but rather moment-to-moment within-person differences. Beyond concurrent emotional states, additional factors such as location, attention, or recent events or stressors may influence individual momentary engagement with skill practice exercises and should be the focus of future research. Additionally, although it might be expected that participants would show higher-quality practice when using their most frequently practiced skill, perhaps due to greater familiarity or benefit from repetition, this was not observed. Instead, participants engaged with their most frequently practiced skill at a quality level comparable to that of their least frequently practiced skills. This finding further highlights the need to explore other external factors that may influence engagement quality and suggests that skill choice does not introduce bias in overall quality ratings. Allowing participants to choose which skill to practice reflects real-world clinical application. However, familiarity with a skill or its frequency of use, compared to other skills, did not translate to higher-quality engagement. Future research may focus on identifying strategies to enhance overall skills practice exercise quality rather than assuming that preference for a skill leads to better engagement.

When comparing across the three skills, CF practice exercises overall received lower quality ratings than MEA and CEB practice exercises. This suggests that participants may have found CF more challenging to engage with effectively. It may be that participants simply did not understand the skill as it was meant to be practiced, thereby influencing quality codes. Alternatively, participants may have perceived the skill as more cognitively effortful compared to the other two skills. This could make high quality, in-the-moment engagement more difficult, particularly in moments of elevated suicidal ideation, as discussed further below. Conversely, it is possible that participants felt able to engage effectively with CF without producing high-quality in-app responses to the exercise. Indeed, the degree to which the quality of EMI submissions reflects the actual extent to which participants practiced any given CBT skill is unknown. Low quality or limited text responses in the application doesn’t necessarily mean the skill wasn’t practiced effectively in the moment. Further research is needed to explain which, if any, of these possible explanations accounts for the lower quality average of in-app CF practice compared to the other two skill exercises. Coding quality of engagement in this way provides a valuable method for assessing the difficulty of different intervention skills, offering insight into how participants engage with various skills across EMI-based interventions even beyond this specific EMI.

While quality averages differed between CF and the other two skills, higher pre-EMI suicidal urge and intent were associated with lower overall skills practice exercise quality across skill types. This supports the idea that suicide-related distress may interfere with one’s ability to fully and relevantly engage with EMI prompts, suggesting that it may be more difficult to practice CBT skills when suicidal thoughts are more intense. In contrast, no measured momentary affect states (i.e., agitated, angry, fatigued, positive, negative, grateful, connected, hopeless, and burdensome) were significantly associated with skills practice exercise quality. This suggests that immediate emotional experiences may be less influential than suicidal ideation in predicting how meaningfully individuals engage with CBT skills practice exercises. These findings may also reflect the clinical reality that, during periods of heightened suicidal thoughts or urges, individuals may struggle to effectively engage with more cognitively demanding skills like those based in CBT. As the current EMI was designed to support CBT skill practice broadly across varying affective states, these results suggest that during moments of increased suicidal thinking, individuals may achieve higher-quality engagement by turning to strategies found in other modalities such as distress tolerance or self-soothing techniques.

Although overall quality assessed across all skills was not associated with proximal changes in suicidal thoughts or affect, analyses of individual skills revealed that higher-quality CEB practice exercises were associated with greater reductions in suicidal intent, and both CF and CEB exercise quality were associated with decreased anger. These findings suggest that quality of engagement with the CEB skill may be particularly important in reducing suicidal thoughts and distress. For lower-quality CEB practice exercises, individuals may not have effectively identified an alternate more helpful behavior, potentially suggesting persistent engagement in identified emotion-driven behaviors or lack of engagement in a more helpful alternative action. Therefore, quality of engagement with the CEB skill exercises may be an important target for clinical work. Clinicians may consider providing patients with concrete examples of alternative behaviors across different emotional contexts or offering guidance on how to differentiate helpful from unhelpful behaviors (e.g., evaluating the short- and long-term consequences of each).

Additionally, quality of engagement with CF and CEB practice exercises was associated with post-practice intensity of anger in particular. This finding suggests that anger may be more sensitive than other emotional states to quality of engagement in these active coping strategies. It is also possible that higher quality engagement is more time-intensive and cognitively effortful than lower quality engagement, thereby allowing anger, which is a high-arousal emotion that can fluctuate quickly, to dissipate over the course of the intervention (Heylen et al., 2015). Though the mechanisms of these findings remain unclear, they suggest that higher quality skill practice with the CF and CEB skill exercises may be particularly important for regulating anger. These findings highlight that understanding how individuals engage with each skill, especially how quality differs across skill and emotional context, helps reveal which distinct affect states each skill is most effective in regulating.

Interestingly, quality of MEA skills practice exercise was not associated with proximal changes in any measured outcomes. This may indicate that the quality of a mindfulness skill is less important for driving immediate emotional change, at least in this high-risk population. Taking a brief moment to practice this skill, regardless of engagement level, may be enough to reduce negative emotions. This aligns with findings from broader mindfulness-based interventions, where brief engagement with mindfulness techniques (e.g., deep breathing, body scans) can been linked to reduced distress post-intervention (Schumer et al., 2018; Balban et al., 2023). However, while prior studies have examined proximal outcomes following mindfulness-based digital interventions, none thus far to our knowledge have investigated the immediate, in-the-moment effects post skill practice on affect and suicidal thoughts delivered via EMI, nor have any examined these immediate effects within populations at elevated risk for suicidal thoughts and behaviors (Mrazek et al., 2019; Pavlacic et al., 2022; Pavlacic et al., 2025; Schmelefske et al., 2022). The null association between MEA practice quality and proximal outcomes may also indicate that the specific mindfulness-based skill in the present study is not effective at reducing proximal negative affect, even when practiced at a high-quality level. Each of these possibilities aligns with findings from broader mindfulness-based intervention research, which report inconsistent evidence for the impact of mindfulness-based interventions and improvement in negative affect post-intervention (Keng et al., 2019). In considering the lack of effect for quality of practice on outcomes in the mindfulness skill, it is possible that the benefits of engaging with MEA exercises at higher quality emerge over a longer time frame rather than immediately following practice. Further research is needed to uncover the role of practice quality in emotional outcomes over time, particularly in the context of in-the-moment digital mindfulness-based interventions.

### 3.1. Limitations

This study has several limitations that should be considered when interpreting the findings. First, operationalizing and coding skill practice exercise quality based on text input comes with challenges. For example, as mentioned earlier, participants may have meaningfully engaged with the skills practice exercise in real time but provided only brief or incomplete responses in the smartphone application. This could have occurred in cases of reluctance to type out their full thoughts or time it took to do so. Additionally, the shorthand nature of some responses made it difficult to accurately assess quality, as it was not always clear what participants intended to convey. Second, although the sample size is small, as a pilot study, these results provide an important foundation for future research but should be replicated in larger samples. Finally, the sample lacked racial and ethnic diversity, which limits the generalizability of the results to broader populations. Future research should aim to include more diverse participants to better understand how skill practice exercise quality varies across different demographic and clinical groups.

### 3.2. Future directions

Future research should expand on these findings in several ways. First, examining the effects of skill practice exercise quality over longer periods of time may provide insight into whether quality has sustained effects on suicidal thoughts and emotion regulation. This may be particularly relevant for CEB, as participants may require more time to fully implement an alternative behavior before experiencing its benefits. Second, replicating these findings in a larger dataset will help determine the robustness of these effects and improve generalizability. Third, future work can investigate whether integrating additional skills into this EMI may lead to higher-quality engagement, particularly during moments of heightened suicidal thoughts or urges. Finally, just-in-time adaptive interventions (JITAIs) are designed to provide support in the moments people need it most (Nahum-Shani et al., 2016). Developing JITAIs that predict real-time engagement quality based on suicidal thinking intensity is a promising next step to enhance the personalization of mobile interventions and improve intervention delivery and efficacy. Similarly, integrating real-time quality assessment into mobile interventions could inform JITAIs aimed at improving quality of skills practice exercises in the moment. This could enable immediate detection of lower-quality responses, triggering supplementary interventions such as psychoeducation, additional skill-practice support, or motivational guidance. Future research should explore whether these mini-supplementary interventions could improve skill engagement quality and, ultimately, intervention efficacy.

### 3.3. Conclusions

These results suggest that the degree to which the quality of in-the-moment EMI-based skills practice is associated with short-term skill effectiveness may differ as a function of both CBT skill and distress type. The quality of skills practice exercises may play a role in intervention efficacy for certain emotion management skills, while others may not rely as much on relevance and completion to be effective. Overall, this study highlights the importance of understanding skill practice quality in the context of in-the-moment, smartphone-based CBT interventions for individuals in periods of high distress. Given the potential for digital interventions to extend the reach of CBT beyond traditional therapy settings, understanding how quality of engagement impacts effectiveness is a critical step toward improving intervention design and delivery. We must continue to better understand the nuances of engagement with smartphone-based interventions and CBT skills practice to bolster the precision and impact of digital intervention tools. Better understanding how quality of engagement impacts emotional outcomes can pave the way for more refined and personalized real-time interventions for suicidal ideation and emotional distress.

## Figures and Tables

**Table 1:** Participant demographic characteristics.

Characteristic	% (n)	M (SD, range)
Age	–	34.4 (14.1, 19–63)
Gender		
Female	57% (13)	–
Male	43% (10)	–
Race		
White	61% (14)	–
Asian	22% (5)	–
Black	8.7% (2)	–
Other	8.7% (2)	–
Ethnicity		
Non-Hispanic	82% (18)	–
Hispanic	18% (4)	–

*Note: N* = 23, except for ethnicity (*n* = 22) and age (*n* = 21).

**Table 2:** Aim 2 multi-level models results: pre-EMI emotional context as predictors of quality. Bolded values indicate statistical significance.

Predictor	Skills practice quality	
	Estimates	*p*
Suicidal intent	−0.06	**0.033** *
Suicidal urge	−0.04	**0.032** *
Positive	0.01	0.285
Negative	0.02	0.196
Grateful	−0.00	0.933
Connected	0.01	0.431
Hopeless	0.01	0.551
Fatigued	0.01	0.248
Angry	−0.02	0.260
Agitated	0.01	0.488
Burdensome	0.02	0.192

Note: Each row represents results from a separate MLM.

* *p* < 0.05.

**Table 3:** Aim 3 multi-level models results: skills practice quality as a predictor of proximal change. Bolded values indicate statistical significance.

	Suicidal intent		Suicidal urge		Grateful	
	Estimates	*p*	Estimates	*p*	Estimates	*p*
Quality_Overall_	0.03	0.448	0.04	0.451	0.03	0.789
Quality_CEB_	0.30	**0.003** **	0.22	0.185	−0.21	0.374
Quality_TF_	−0.01	0.842	0.02	0.805	−0.02	0.869
Quality_MEA_	−0.06	0.408	−0.04	0.595	0.20	0.235
	Connected		Hopeless		Fatigued	
	Estimates	*p*	Estimates	*p*	Estimates	*p*
Quality_Overall_	−0.05	0.585	−0.06	0.538	−0.01	0.953
Quality_CEB_	−0.31	0.260	1.22	0.306	0.32	0.691
Quality_TF_	0.06	0.673	0.00	0.975	−0.17	0.236
Quality_MEA_	−0.07	0.689	−0.17	0.425	0.00	0.990
	Angry		Agitated		Burdensome	
	Estimates	*p*	Estimates	*p*	Estimates	*p*
Quality_Overall_	0.12	0.364	−0.06	0.406	−0.19	0.178
Quality_CEB_	0.49	<0.001***	0.06	0.746	0.14	0.532
Quality_TF_	0.31	**0.006** **	−0.10	0.325	0.02	0.916
Quality_MEA_	−0.20	0.222	−0.05	0.733	−0.42	0.058

Note: Each cell of the table represents results from a separate MLM.

** *p* <0.01.

*** *p* <0.001.

## Appendix B. EMI Quality Rating Scale

### Mindful Emotional Awareness (MEA)

Rating 1 (three-point check in): Rate how well the participant differentiated between components of their current emotional experience (thoughts, physical feelings, behaviors/urges, emotions).

**0** Missing or extremely limited: Missing (or functionally missing; e.g., “nothing”) responses in all three text boxes OR only one component of emotional experience identified across the three text boxes; for example, only recording one emotion [e.g., “angry”], thought [e.g., “my life sucks”], feeling [e.g., “tired”], or behavior/urge [e.g., “sitting”] would be rated 0.

**1** Somewhat relevant or complete: Two components of emotional experience (thoughts, physical feelings, behaviors, or emotion) are entered across the three text boxes, regardless of whether the component is entered in the “correct” box (e.g., if the participant appropriately identifies a thought and a physical feeling, rate a 1 even if one or both are entered in the “incorrect” box). Note that entering an emotion (e.g., “sad”) in one or more of the text boxes is acceptable, as long as at least one thought, physical feeling, or behavior/urge is also identified in another box. Can also rate 1 if the response doesn’t otherwise meet criteria for 0 or 2.

**2** Highly relevant and complete: At least three distinct components of emotional experience (thoughts, physical feelings, behaviors, or emotion) are entered across the three text boxes, regardless of whether the component(s) is entered in the right box. If one of the components is an emotion, rate 2 as long as the other two components identified contain thoughts, physical feelings, or behaviors/urges.

Rating 2 (assessment of present focus): Rate whether (and how well) the participant assessed if their thoughts, feelings, and behaviors are in line with the present, past, or future.

**0** Missing or extremely limited: None of the three questions about focus on present, past, or future are answered, OR the response(s) are clearly not aligned with the content entered in the three-point check (e.g., participant entered a thought that is clearly about the past in the three-point check, but said “no” to thinking about something that happened in the past [and they either did not answer the other two questions or did not give at least one other clearly consistent response]).

**1** Somewhat relevant or complete: One or two of the three questions assessing present, past, or future focus are answered AND at least one of the responses appears consistent with the content entered for the three-point check, OR the participant responded to all three questions, but one or two of the responses is clearly inconsistent with the content entered for the three-point check. Can also rate 1 if practice doesn’t otherwise meet criteria for 0 or 2.

**2** Highly relevant and complete: All three questions assessing present, past, and future focus are answered AND there are no obvious inconsistencies with what is entered for the three-point check.

### Cognitive Flexibility (CF)

Rating 1 (identification of automatic negative thoughts): Rate whether and how well the participant identified at least one negative thought likely tied to strong levels of emotion.

**0** Missing or extremely limited: Missing (or functionally missing; e.g., “nothing”) response OR response is blatantly irrelevant (e.g., “I’m working”) OR clearly a positive thought (e.g., “things are good today”).

**1** Somewhat relevant or complete: Not clearly an automatic negative thought but reasonable to assume that the response is shorthand for a negative or neutral thought (e.g., “about my mom”), OR response is better categorized as a maladaptive emotion-driven behavior (e.g., “going to bed”), emotion (e.g., “angry”), or physical sensation (e.g., “restless”) tied to emotion. Can also rate 1 if practice doesn’t otherwise meet criteria for 0 or 2.

**2** Highly relevant and complete: Participant clearly identified one or more automatic negative thought(s), even if shorthand is used (e.g., “life sucks,” “never get better,” “ending my life”).

Rating 2 (generation of alternative thoughts): Rate whether and how well the participant came up with at least one other interpretation for the automatic negative thought entered.

**0** Missing or extremely limited: Missing (or functionally missing; e.g., “nothing”) response OR entirely irrelevant (e.g., “going to work”) OR a maladaptive behavior or urge (e.g., “want to hurt self”) OR response is simply another way of stating the initial automatic negative thought.

**1** Somewhat relevant or complete: Not clearly an alternative thought but reasonable to assume that the response is shorthand for another interpretation (e.g., response is “yes” when “Could there be any other explanations?” was selected), OR response is better categorized as a new, more adaptive/change-oriented behavior (e.g., “try exercising”), emotion, or physical sensation that is relevant to (i.e., a change from) the initial automatic negative thought (e.g., “feel calm” if thought was anxiety-related), OR response is an adaptive or neutral thought but clearly unrelated to initial automatic negative thought. Can also rate 1 if practice doesn’t otherwise meet criteria for 0 or 2.

**2** Highly relevant and complete: Participant clearly identified one or more alternative interpretations for their initial automatic negative thought, even if shorthand was used (e.g., “that may not happen,” “feels like it but isn’t true,” “not for certain”).

### Countering Emotional Behavior (CEB)

Rating 1 (identification of unhelpful emotional behaviors/urges): Rate whether and how well the participant identified at least one unhelpful behavior or urge likely tied to strong emotion.

**0** Missing or extremely limited: Missing (or functionally missing; e.g., “nothing”) response OR response is blatantly irrelevant (e.g., “good”) OR clearly an adaptive or helpful behavior (e.g., “stay safe”).

**1** Somewhat relevant or complete: Response may be better categorized as an automatic negative thought (e.g., “what’s the point”), emotion (e.g., “angry”), or physical sensation (e.g., “tense”) tied to emotion but is clearly a component of emotional experience. Can also rate 1 if practice doesn’t otherwise meet criteria for 0 or 2.

**2** Highly relevant and complete: Participant clearly identified one or more unhelpful emotional behavior.

Rating 2 (generation of alternative actions): Rate whether and how well the participant came up with at least one more adaptive behavior for the emotional behavior entered.

**0** Missing or extremely limited: Missing (or functionally missing; e.g., “nothing”) response OR entirely irrelevant (e.g., “going to work”) OR response is clearly still negative or simply another way of stating the initial unhelpful emotional behavior.

**1** Somewhat relevant or complete: Not clearly an alternative action/behavior but reasonable to assume that the response is shorthand for one (e.g., “run”) OR response is better categorized as a new, more adaptive/change-oriented thought (e.g., “it will be okay”), emotion, or physical sensation that is relevant to the initial emotional behavior (e.g., “it will be okay” if initial behavior was “avoid/stay in bed”). Can also rate 1 if practice doesn’t otherwise meet criteria for 0 or 2.

**2** Highly relevant and complete: Participant clearly identified at least one clearly more adaptive behavior/action that appears relevant to the initial emotional behavior.
